# A case of reactive lymphoid hyperplasia of the liver in a patient with autoimmune hepatitis

**DOI:** 10.1186/s40792-020-00856-3

**Published:** 2020-05-04

**Authors:** Hiroki Kanno, Hisamune Sakai, Toru Hisaka, Satoki Kojima, Ryuta Midorikawa, Shogo Fukutomi, Yoriko Nomura, Yuichi Goto, Toshihiro Sato, Munehiro Yoshitomi, Ryuichi Kawahara, Koji Okuda

**Affiliations:** Department of Surgery, Kurume University School of Medicine, 67 Asahi-machi, Kurume, Japan

**Keywords:** Reactive lymphoid hyperplasia, Liver, Autoimmune hepatitis

## Abstract

**Background:**

Reactive lymphoid hyperplasia (RLH) of the liver is a benign disorder. It is usually observed in the skin, orbit, thyroid, lung, breast, or gastrointestinal tract, but rarely in the liver. Since the first report of RLH of the liver in 1981, only 75 cases have been described in the past literature. Herein, we report a case of RLH of the liver in a patient with autoimmune hepatitis (AIH), which was misdiagnosed as hepatocellular carcinoma (HCC) preoperatively and resected laparoscopically.

**Case presentation:**

A 43-year-old Japanese woman with autoimmune hepatitis was followed up for 5 years. During her medical checkup, a hypoechoic nodule in segment 6 of the liver was detected. The nodule had been gradually increasing in size for 4 years. Abdominal ultrasound (US) revealed a round, hypoechoic nodule, 12 mm in diameter. Contrast-enhanced computed tomography (CT) demonstrated that the nodule was slightly enhanced in the arterial dominant phase, followed by perinodular enhancement in the portal and late phases. A magnetic resonance imaging (MRI) scan showed low signal intensity on the T1-weighted image (T1WI) and slightly high signal intensity on the T2-weighted image (T2WI). The findings of the Gd-EOB-DTPA-enhanced MRI were similar to those of contrast-enhanced CT. Tumor markers were all within the normal range. The preoperative diagnosis was HCC and a laparoscopic right posterior sectionectomy was performed. Pathological examination revealed that the nodular lesion was infiltrated by small lymphocytes and plasma cells, and germinal centers were present. Immunohistochemistry was positive for B cell **and T cell markers, indicating polyclonality.** The final diagnosis was RLH of the liver.

**Conclusions:**

The pathogenesis of RLH of the liver remains unknown, and a definitive diagnosis based on imaging findings is extremely difficult. If a small, solitary nodule is found in female patients with AIH, the possibility of RLH of the liver should be considered.

## Background

Reactive lymphoid hyperplasia (RLH), also called pseudolymphoma or nodular lymphoid lesions, of the liver is a benign disorder. It is characterized by a marked proliferation of non-neoplastic polyclonal lymphocytes forming follicles with active germinal centers. RLH is usually observed in the skin, orbit, thyroid, lung, breast, or gastrointestinal tract, but rarely in the liver. Since the first report of RLH of the liver in 1981 by Snover et al. [1], only 75 cases have been described in the past literature. Etiology and pathogenesis of RLH of the liver is completely unknown.

Herein, we report a case of RLH of the liver in a patient with autoimmune hepatitis (AIH), which was misdiagnosed as hepatocellular carcinoma (HCC) preoperatively and resected laparoscopically.

## Case presentation

A 43-year-old Japanese woman was followed for AIH for 5 years and has been prescribed oral steroids. She had a medical history of cerebral hemorrhage. During her medical checkup, a hypoechoic nodule in segment 6 (S6) of the liver was detected by abdominal ultrasound (US). It was initially diagnosed as a simple cyst. The nodule gradually increased from 7 mm to 12 mm in diameter over a 4-year period. On physical examination, there were no abnormalities. A laboratory examination showed no remarkable abnormalities: aspartate aminotransferase 19 U/L, alanine aminotransferase 19 U/L, alkaline phosphatase 168 U/L, γ-glutamyltransferase 11 U/L, total bilirubin 0.5 mg/dl, albumin 3.90 g/dl, platelet counts 32.1 × 10^4^/μl, and prothrombin time 113%. Serum IgG level was 2566 mg/dl. Tumor markers were within normal range: α-fetoprotein 1.2 ng/ml, protein-induced vitamin K absence-II 16.0 mAU/ml, carcinoembryonic antigen 0.3 ng/ml, and carbohydrate antigen 19-9 8.1 U/ml. Hepatitis virus markers were all negative.

**Fig 1:**
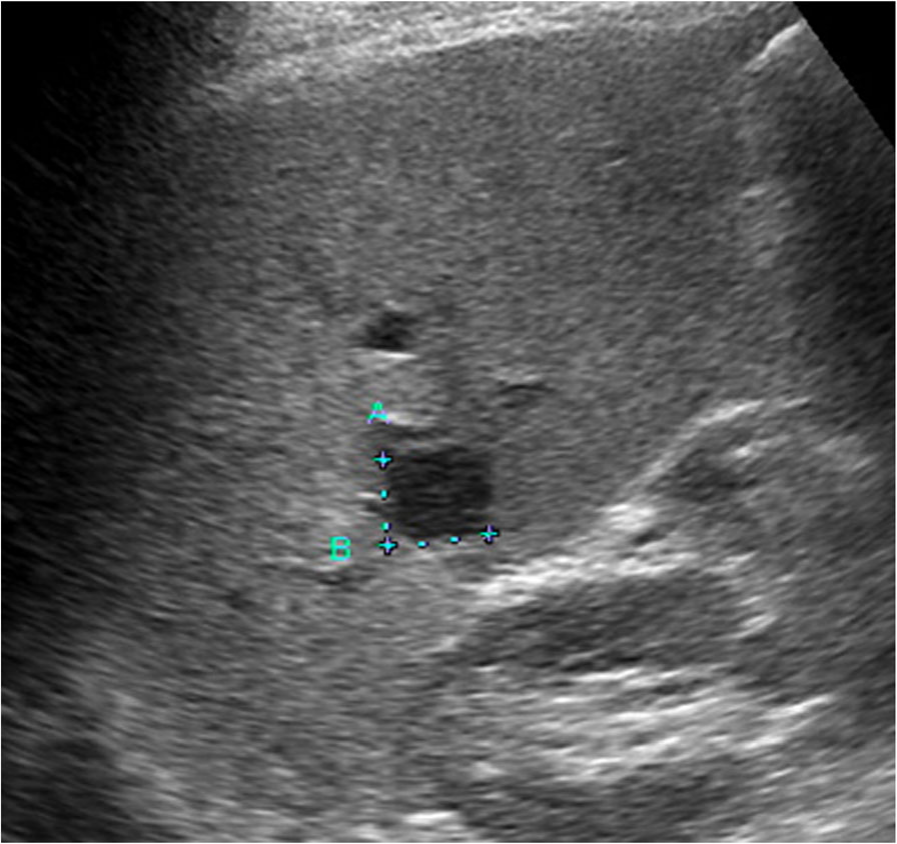
Abdominal ultrasound reveals a well-demarcated, homogenous, hypoechoic round nodule in segment 6 of the liver

**Fig. 2:**
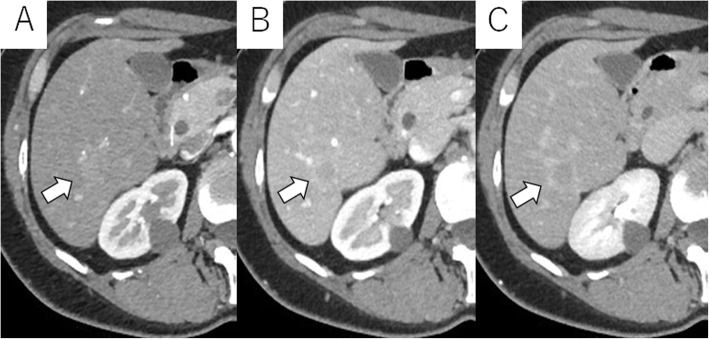
On CT, the nodule is slightly enhanced in the arterial dominant phase (**a**). The lesion shows perinodular enhancement in the portal and delayed phases (**b**, **c**) (white arrow)

**Fig. 3:**
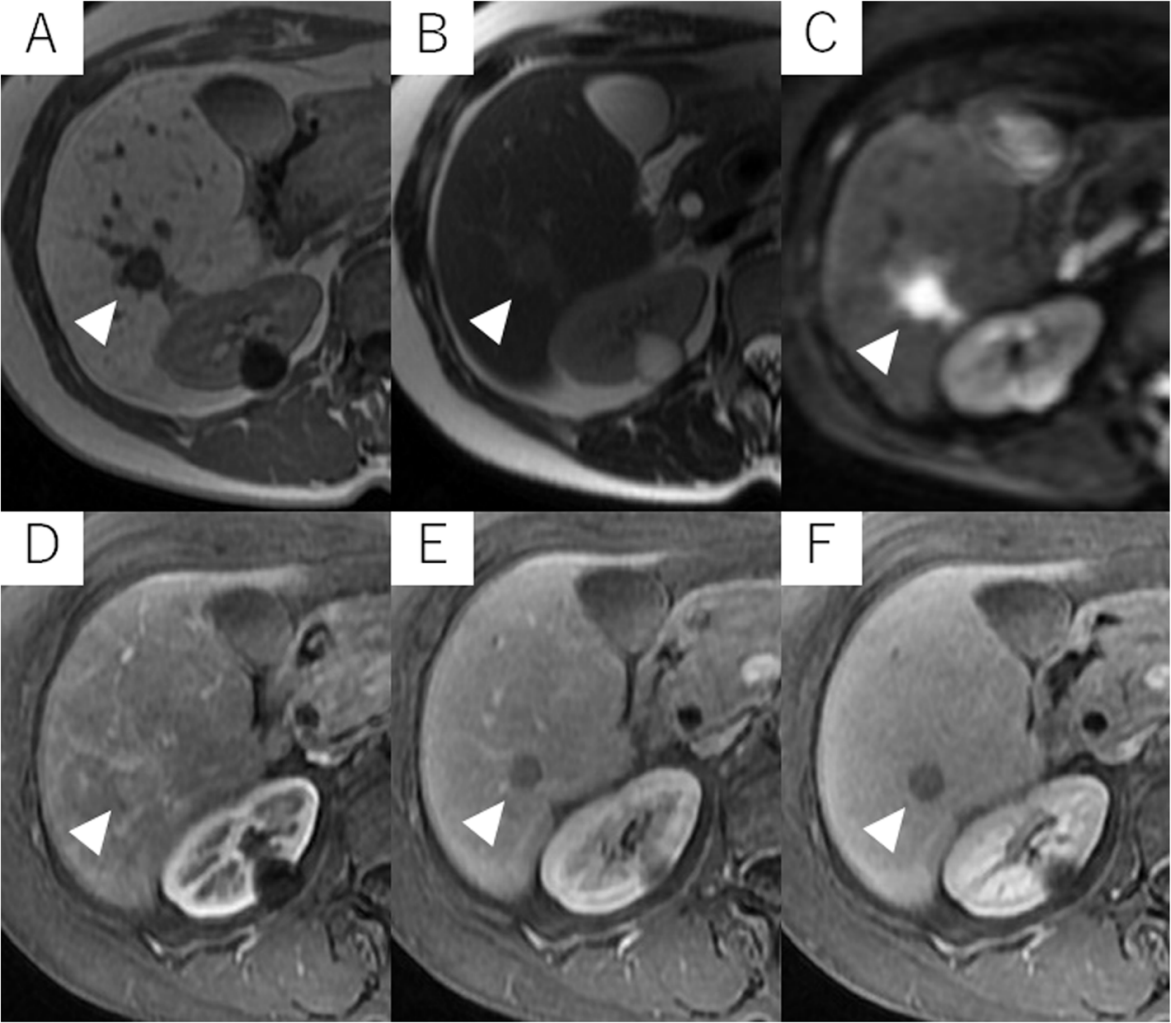
MRI demonstrates that the nodule shows well-defined low signal intensity in T1-weighted image (**a**), slightly high signal intensity in T2-weighted image (**b**), and strongly high signal intensity in diffusion-weighted image (**c**). Gd-EOB-DTPA-enhanced MRI reveals that the nodule is slightly enhanced in the arterial phase (**d**). The lesion shows perinodular enhancement in the portal phase (**e**) and a defect in hepatobiliary phase (**f**) (white arrowhead)

Abdominal US revealed a round hypoechoic homogeneous nodule, 12 mm in diameter (Fig. 1). It was enhanced in the arterial dominant phase followed by defected in the post vascular phase by the contrast medium. Plain computed tomography (CT) showed low density in S6 of the liver and contrast-enhanced CT demonstrated that the nodule was slightly enhanced in the arterial dominant phase followed by perinodular enhancement in the portal and late phases (Fig. 2). Magnetic resonance imaging (MRI) showed low signal intensity on the T1-weighted image (T1WI) and slightly high signal intensity on the T2-weighted image (T2WI). On the diffusion-weighted image (DWI), the lesion showed high signal intensity. Gadolinium ethoxybenzyl diethlenetriamine pentaacetic acid (Gd-EOB-DTPA)-enhanced MRI showed similar findings as contrast-enhanced CT and subsequently presented the defect in the hepatobiliary phase (Fig. 3).

The preoperative diagnosis was HCC. Because there was a risk of intraabdominal seeding, we did not perform percutaneous needle biopsy of the nodule. The patient received laparoscopic right posterior sectionectomy. The cut section of the resected liver showed a well-demarcated, round, yellowish-white lesion measuring about 14 mm.

**Fig. 4:**
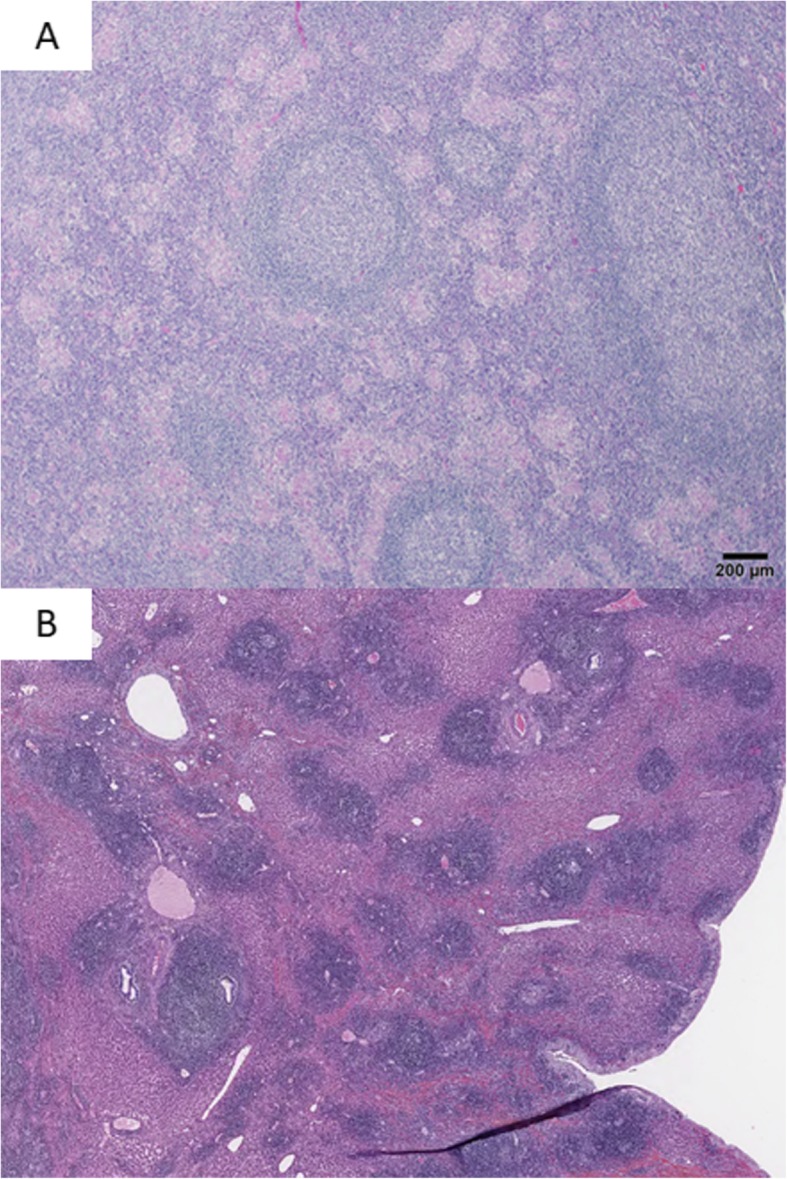
Pathological examination reveals that germinal centers are present and small lymphocytes and plasma cells are infiltrating the lesion (**a**). Marked lymphoid cell infiltration in the portal tracts was observed in the perinodular lesion (b)

**Fig. 5:**
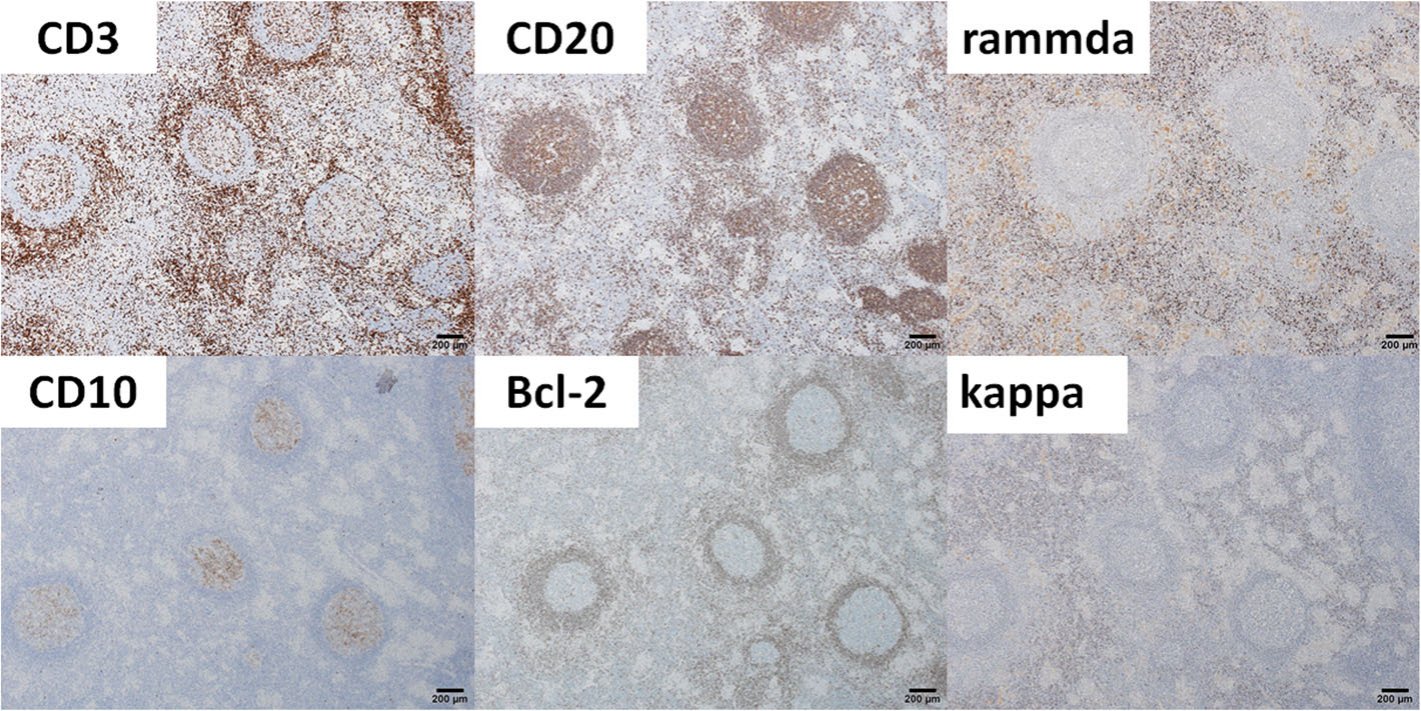
Immunohistochemical staining reveals that the follicles are CD20-positive. The lymphocytes in the germinal centers are CD10-positive and Bcl-2-negative. The interfollicular area is composed of CD3-positive small T cells. Infiltrating plasma cells show a polyclonal expression of cellular immunoglobulin kappa and lambda chains

Pathological examination revealed a nodular lesion without fibrous capsule. The lesion was infiltrated by small lymphocytes and plasma cells, and germinal centers were present (Fig. 4a). Marked lymphoid cell infiltration was found in the portal tracts around the nodule (Fig. 4b). Although many small granulomas were found in the lesion, infectious pathogens were not found on periodic acid-Schiff, Gomori-Grocott, or Ziehl-Neelsen stainings. Immunohistochemical staining revealed that the follicles were CD20-positive. The lymphocytes in the germinal centers were CD10-positive and Bcl-2-negative. The interfollicular area was composed of CD3-positive small T cells. Infiltrating plasma cells showed a polyclonal expression of cellular immunoglobulin kappa and lambda chains (Fig. 5). Some IgG4-positive plasma cells were present, but most were IgG-positive plasma cells. The final diagnosis was RLH of the liver.

The post-operative course was uneventful and she was discharged on post-operative day nine.

## Discussion

RLH of the liver is rare. Based on a review of the PubMed database from 1981 to 2019 using the keywords “Reactive lymphoid hyperplasia,” “Pseudolymphoma,” and “Liver,” we found 75 cases of RLH of the liver [1–51]. These cases, along with our case, are summarized in Table 1. Of the 76 cases, 71 (93.4%) were female. The average patients’ age was 56.7 years old (range, 15–85). Among them, 35.5% had liver diseases, 17.1% had autoimmune disease, and 27.6% had a malignant tumor. Many were solitary (84.7%), the average size being 16.3 mm (range, 4–105 mm) and 81.3% of the tumors were less than 20 mm in diameter. Many were located in the right lobe (62.9%). 93.1% were diagnosed as malignant tumors such as HCC, cholangiocellular carcinoma (CCC), or metastatic. Surgical resection was performed in 82.9% of cases. US findings showed most of the lesions were round and hypoechoic. Plain CT revealed low density and findings on contrast-enhanced CT ranged from enhanced to slightly enhanced to perinodular enhancement. On MRI, almost all lesions were found to have low intensity on T1WI and high intensity on T2WI and presented various enhancement patterns similar to the CT findings.

**Table 1:** Clinicopathological features of reactive lymphoid hyperplasia of the liver

Variables	*n* = 76	Percentage
Clinical characteristics		
Age (ave, range)	56.7 (15–85)	
Sex		
Male	5	6.6
Female	71	93.4
Liver diseases		35.5
Viral hepatitis B	6	
Viral hepatitis C	2	
Primary biliary cirrhosis	14	
Non-alcoholic steatohepatitis	2	
Autoimmune hepatitis	3	
Autoimmune disorders		17.1
Chronic thyroiditis	4	
Sjögren’s syndrome	3	
Rheumatoid arthritis	1	
Takayasu disease	1	
Antiphospholipid syndrome	1	
CREST syndrome	1	
Immunodeficiency	2	
Malignant tumor		27.6
Colon cancer	6	
Gastric cancer	5	
Renal cell carcinoma	4	
Cervical cancer	1	
Thyroid cancer	1	
Bile duct cancer	1	
Pancreatic cancer	1	
Ovarian cancer	1	
GIST	1	
Number of nodules		
Solitary	61	84.7
Multiple	11	15.3
Nodule size (ave, range) (mm)		
< 20	65	81.3
≥ 20	15	18.7
Location		
Rt. lobe	39	62.9
Lt. lobe	15	24.2
Caudate lobe	3	4.8
Bilobar	5	8.1
Preoperative diagnosis		
Hepatocellular carcinoma	31	53.4
Cholangiocellular carcinoma	4	6.9
Metastatic	12	20.7
Malignant tumor	7	12.1
Others	4	6.9
Treatment		
Operation	63	82.9
Biopsy	8	10.5
Transplantation	3	3.9
Others	2	2.6
Imaging findings (described)		
US		
Hypo	39	51.3
Iso	1	1.3
Unknown	36	47.4
CT (plain)		
Low	29	38.2
Not detected	2	2.6
Unknown	45	59.2
CT (arterial phase)		
Enhanced	15	19.7
Slightly enhanced	15	19.7
Perinodular enhancement	9	11.8
Not enhanced	5	6.6
Unknown	32	42.1
CT (portal or delay phase)		
Washout	23	30.3
Perinodular enhancement	8	10.5
Not enhanced	4	5.3
Others	5	6.6
Unknown	36	47.4
MRI (T1WI)		
Hypo	42	55.3
Slightly hypo	4	5.3
Unknown	30	39.5
MRI (T2WI)		
Hyper	38	50.0
Slightly hyper	5	6.6
Iso	3	3.9
Unknown	30	39.5
MRI (arterial phase)		
Enhanced	13	17.1
Slightly enhanced	14	18.4
Perinodular enhancement	8	10.5
Others	3	3.9
Unknown	38	50.0
MRI (portal or delay phase)		
Washout	19	25.0
Perinodular enhancement	8	10.5
Others	3	3.9
Unknown	46	60.5
PET		
Positive	8	10.5
SUV max (ave, range, *n* = 6)	4.9(3.4–6.7)	
Negative	2	2.6
Unknown	66	86.8

Etiology and pathogenesis of RLH of the liver is not fully understood. Many patients with RLH of the liver have liver disease, autoimmune disease, or a malignant tumor. It is speculated that RLH is related to a reactive immunological response to a chronic inflammatory process. Nakabayashi et al. reported that tumor growth factor-β knockout mice showed multiple lymphoproliferative disorders similar to those observed in the pseudolymphoma of Sjögren’s syndrome, and that upregulation of several inflammatory cytokine genes, interferon-γ, interleukin (IL)-1, IL-6, and IL-10, was observed in their salivary glands [52]. Up/downregulation of some cytokines and chemokines are strongly associated with cancer growth and progression [53]. Many cases of RLH of the liver have a history of malignant tumors; cytokines and chemokines derived from the malignant tumors may affect the occurrence of RLH.

The imaging findings of RLH are nonspecific and varied from strongly enhanced, slightly enhanced, to perinodularly enhanced on contrast CT and MRI. Thus, it is extremely difficult to differentiate hepatic RLH from malignant tumors such as HCC and CCC and metastatic tumors based on imaging findings alone. Indeed, surgical resections were performed for the diagnosis of these malignancies in many cases. The nodule increased in size in some cases, including ours [8, 34, 48], but in other cases, it reduced without any treatment [14, 27]. Additionally, malignant conversion has been reported in the lungs, stomach, and skin [54–56]. In RLH of the liver, only one case transformed into mucosa-associated lymphoid tissue lymphoma [57]. Therefore, we think that pathological diagnosis by needle biopsy and intensive follow-up is warranted.

Histologically, RLH of the liver consists of hyperplastic lymphoid follicles, lymphocytes, and other inflammatory cells. Immunohistochemical staining is positive for B cell markers (CD20 or CD79α) and T cell markers (CD3 or CD4), indicating polyclonality. CD20-positive B cells are predominantly located in the lymphoid follicles and CD3-positive T cells are predominantly located in interfollicular areas. Additionally, the lymphocytes in the germinal centers are negative for Bcl-2, which indicates the non-neoplastic and reactive nature [30]. Some reported that portal venular disappearance and/or stenosis, caused by marked lymphoid cell infiltration in the portal tracts around nodules, was observed in the perinodular lesion [22, 27, 31, 57, 58]. These pathological changes may induce portal flow disturbances and increased hepatic arterial flow in the perinodular lesion, resulting in perinodular enhancement. A similar finding was found in the present case.

In a meta-analysis [59], the pooled HCC incidence rate in AIH patients without cirrhosis was 1.14 per 1000 person-years and 10.07 per 1000 person-years in AIH patients with cirrhosis, which was lower than viral hepatitis such as hepatitis B or C. When a nodule is detected in a patient with AIH, we should consider the possibility of RLH of the liver.

## Conclusions

We presented a case of RLH of the liver associated with AIH and performed a sectionectomy with the presumptive diagnosis of HCC. However, the lesion was found to be a benign RLH upon pathological and histochemical examination of the resected specimen. The pathogenesis of RLH of the liver remains unknown, and a definitive diagnosis based on imaging findings is extremely difficult. If a small, solitary nodule is found in female patients with AIH, the possibility of RLH of the liver should be considered.

## Data Availability

Not applicable

## Abbreviations

AIH: Autoimmune hepatitis
CT: Computed tomography
DWI: Diffusion-weighted image
Gd-EOB-DTPA: Gadolinium ethoxybenzyl diethlentriamine pentaacetic acid
HCC: Hepatocellular carcinoma
MRI: Magnetic resonance imaging
RLH: Reactive lymphoid hyperplasia
T1WI: T1-weighted image
T2WI: T2-weighted image
US: Ultrasonography
